# Enhancing Well-Being in Cancer Survivors Through Acceptance and Commitment Therapy: A Randomized Clinical Trial

**DOI:** 10.7759/cureus.99646

**Published:** 2025-12-19

**Authors:** Antonios Bozas, Anna Nisyraiou, Maria Vasilopoulou, Konstantina Stavrogianni, Marianna Zacharia, Maria Karekla, Mara Gkioka, Christina Karamanidou

**Affiliations:** 1 Institute of Applied Biosciences, Centre for Research and Technology Hellas, Thessaloniki, GRC; 2 ACThealthy Laboratory, Department of Psychology, University of Cyprus, Nicosia, CYP

**Keywords:** act, cancer survivorship, post-traumatic growth, psychological flexibility, psycho-oncology, randomized controlled trial

## Abstract

Introduction: Cancer affects not only physical health but also leads to considerable psychological difficulties for patients. A subset of survivors’ experiences post-traumatic growth (PTG), reflecting positive psychological changes following trauma. Despite increasing interest, the mechanisms and interventions that enhance PTG are not well understood. This randomized controlled trial (RCT) aims to evaluate the efficacy of an Acceptance and Commitment Therapy (ACT)-based group intervention in fostering PTG in cancer patients diagnosed with breast, head and neck, or colorectal cancer across major oncology centers in northern Greece.

Methodology: In addition to PTG, the trial assesses related psychological and physiological factors, including anxiety, depression, illness perception, spiritual well-being, and stress-related biomarkers, to explore the potential mechanisms and broader outcomes of ACT. The study will utilize a parallel-group design, comparing the ACT intervention with standard care. Participants, that is, patients who have completed their oncological therapy, will be randomized and engaged in six weeks of group sessions, with outcomes assessed through standardized questionnaires (Post-traumatic Growth Inventory (PTGI), Functional Assessment of Chronic Illness Therapy-Spiritual Well-being (FACIT-Sp), Impact of Event Scale-Revised (IES-R), Hospital Anxiety and Depression Scale (HADS)), measuring post-traumatic growth, clinical symptoms, spirituality, and biomarkers. The protocol was developed in accordance with the SPIRIT (Standard Protocol Items: Recommendations for Interventional Trials) 2025 guidelines.

Discussion: By examining the impact of the ACT-based intervention, this study seeks to elucidate the therapeutic approaches that can foster PTG, ultimately enhancing empowerment in cancer survivors. The findings will contribute to the growing body of literature on PTG, informing clinical practice and the development of evidence-based interventions.

## Introduction

Cancer remains a significant health issue worldwide, with millions of new cases diagnosed each year [1]. The impact of cancer extends beyond physical symptoms, often resulting in significant psychological and social challenges for patients [1]. For some individuals, the cancer experience may lead to emotional and psychological difficulties, such as anxiety, depression, and post-traumatic stress disorder (PTSD) [2,3]. However, in recent years, a growing body of research has focused on the potential for positive psychological outcomes following trauma, particularly in the context of cancer. This phenomenon, referred to as post-traumatic growth (PTG), reflects the positive psychological changes that some individuals experience in response to traumatic life events [4]. Rather than simply returning to baseline functioning, many people describe emerging from adversity with a deeper appreciation of life, strengthened personal resilience, more meaningful relationships, and a clearer sense of values and priorities. PTG may also involve the discovery of new possibilities, shifts in identity, and enhanced spiritual or existential understanding. These changes do not negate the distress associated with trauma but reflect the capacity for personal transformation that can occur when individuals engage in reflection, meaning-making, and adaptive coping during recovery [4-7].

While approximately 20.5% of cancer patients demonstrate moderate to high levels of PTG [5,7], the mechanisms that foster this growth remain unclear, and evidence-based interventions to enhance PTG are lacking. Factors such as optimism, healthier lifestyle habits, social support, and time since diagnosis are associated with increased PTG in cancer patients [6-8]. However, most studies have employed cross-sectional designs, which limit the conclusions that can be drawn about the developmental trajectory of PTG [9]. Meta-analyses further highlight the lack of consensus regarding its clinical predictors and mediators, underscoring the need for further research [10].

Although PTG is increasingly recognized as an important outcome in cancer survivorship, significant research gaps remain. Most studies rely on cross-sectional designs, limiting causal inferences about the developmental trajectory of PTG and the factors that actively facilitate it [9,10]. Meta-analyses highlight inconsistent evidence regarding predictors and mechanisms, indicating the need for methodologically rigorous trials [6,9]. No existing studies have simultaneously examined psychological, behavioral, and physiological indicators to elucidate underlying mechanisms. These gaps underscore the necessity for controlled, mechanism-focused research to clarify how targeted interventions can reliably enhance PTG and support adaptive survivorship.

Given the increasing recognition of the potential benefits of PTG for cancer patients, understanding the therapeutic approaches that can facilitate PTG is crucial. Several psychotherapeutic interventions, as third-wave therapies, have shown promise in enhancing PTG in cancer populations [11-13]. Approaches such as Acceptance and Commitment Therapy (ACT), Mindfulness-Based Stress Reduction (MBSR), Compassion-Focused Therapy (CFT), and meaning-centered or mindfulness-based interventions support patients in processing distress, fostering psychological flexibility, clarifying values, and cultivating acceptance and self-compassion. These therapeutic mechanisms appear to facilitate the cognitive and emotional shifts that underlie PTG, helping cancer survivors develop greater resilience, a renewed sense of purpose, and more meaningful engagement with life despite ongoing challenges [11-13]. While several factors are integrated in therapeutic approaches, such as mindfulness, that have shown promise in fostering PTG, trials to assess their impact are sparse [12]. Therefore, there remains the need for empirical studies to evaluate the efficacy of interventions aimed at promoting PTG. Among the emerging therapeutic approaches, ACT is a transdiagnostic, process-based intervention designed to enhance psychological flexibility ability to remain present and act in accordance with personal values despite distressing experiences. This approach has been shown to support improvements in overall functioning and quality of life, particularly among individuals facing life-threatening or chronic health conditions [14,15]. Rather than focusing on symptom elimination, ACT emphasizes behavioral change and adaptive management of internal experiences, helping individuals live meaningfully despite distress [16]. By cultivating acceptance, mindfulness, cognitive defusion, and values-based action, ACT targets key mechanisms of psychological change, enabling patients to engage flexibly with difficult emotions and thoughts instead of avoiding them [17]. In oncology settings, ACT is increasingly recognized as a promising intervention for promoting psychological well-being among cancer patients. Through fostering a compassionate and flexible relationship with suffering, ACT has demonstrated benefits for emotional well-being and may also positively influence physical outcomes such as fatigue and inflammation [18,19].

Recent reviews and clinical trials consistently report that ACT interventions improve psychological flexibility while reducing anxiety, depression, and psychological distress [17]. Evidence from studies involving patients with advanced cancer further highlights ACT’s efficacy in enhancing hope, spiritual well-being, and overall mental health [20]. ACT has been successfully applied in diverse cancer populations, including interventions addressing body image distress among head and neck cancer patients, enhancing resilience and managing chemotherapy side effects in gastric cancer patients when combined with personalized nutrition, and improving self-reported outcomes via online delivery models for broader accessibility [19,21,22]. While these findings are encouraging, some areas-such as ACT’s impact on physical symptoms like pain and fatigue-warrant further exploration [18,23]. Overall, ACT-based interventions appear to offer valuable benefits across multiple domains of cancer survivorship care, representing an important addition to psycho-oncological support strategies.

Given that PTG encompasses emotional, cognitive, and existential shifts and is influenced by multiple psychological and physiological factors, ACT may promote PTG by enhancing psychological flexibility, which has been linked to lower distress and greater well-being. Furthermore, outcomes such as spiritual well-being, illness perception, and reduced anxiety and depression act as both predictors and consequences of PTG, while stress-related biomarkers, including cortisol, reflect physiological changes that may mediate the relationship between psychological flexibility and growth [17,18]. Taken together, these considerations, the current randomized clinical trial (RCT) aims to systematically evaluate the efficacy of an ACT-based intervention in fostering PTG in cancer patients with breast, colon, or head and neck cancer. and to explore the psychological and physiological mechanisms underlying this process. While all three groups demonstrate the potential for PTG, evidence suggests that breast and colorectal cancer survivors often report moderate to high PTG levels, whereas head and neck cancer survivors may show more variable or lower PTG, likely due to greater functional and social disruption associated with their treatments. However, direct comparisons between cancer types remain limited, and current evidence does not support definitive differences among breast, colorectal, and head and neck cancer survivors [17-21].

Specifically, the intervention is expected to empower patients, enhance PTG and spiritual well-being, and reduce anxiety, depression, illness perception, and stress-related biomarkers. In addition, the effectiveness of the ACT-based intervention is expected to reveal the mechanisms through which personal growth develops in cancer patients. The findings will inform the development of targeted, evidence-based interventions to enhance well-being and resilience in oncology care. Through this trial, the study aims to establish an evidence-based intervention that could serve as a benchmark for future studies, guiding the development of more targeted therapies.

## Materials and methods

Study design

The present study will be an RCT enrolling cancer patients diagnosed with breast cancer, head and neck cancer, or colon cancer from major oncology centers in northern Greece. This study will use a parallel group, randomized controlled trial design with a superiority framework to assess whether ACT is superior to usual care in fostering PTG. The study involves participants engaging in an ACT-based intervention aimed at enhancing PTG and quality of life. Participants randomized to the control group will receive usual care, which in this context refers to routine medical oncology follow-up, including standard physician visits, medical monitoring, and access to general hospital-based services. However, the study team will not provide any structured psychological support or psychotherapeutic intervention during the intervention period. The rationale for selecting usual care as the control group lies in the recognition that PTG often occurs naturally in cancer patients [24]. To monitor possible external influences, all participants (both intervention and control groups) will be asked to report any concurrent psychological or psychosocial support they are receiving during the study (e.g., individual psychotherapy, participation in support groups). This information will be recorded at baseline and follow-up and used in exploratory analyses to assess potential confounding effects. Due to the nature of the intervention, blinding is not feasible, as participants will be aware of their group allocation (intervention or control). The intervention, lasting six weeks, consists of 90-minute group sessions (one per week) that will be facilitated by a trained ACT. Cortisol levels, psychological assessments, and questionnaires will be used as outcome measures (pre- and post-assessment). The primary goal is to evaluate the efficacy of ACT in fostering PTG and improving the overall psychological well-being of cancer patients. To minimize differential attention effects between groups, participants in the control arm will receive brief check-ins at the same frequency as the intervention sessions. These check-ins will be limited to neutral, non-therapeutic contacts (e.g., confirming well-being, reminding participants of assessment dates) and will not include any psychological guidance or intervention elements. The study protocol was developed in accordance with the SPIRIT (Standard Protocol Items: Recommendations for Interventional Trials) 2025 guidelines to ensure comprehensive and transparent reporting of all methodological elements of the trial [25,26].

Primary objective

The primary objective of this study protocol, titled “A Manualized Post-Traumatic Growth Intervention for People with Cancer in Greece (IC-Growth) is to assess the efficacy of a determining ACT-based intervention, adapted from the I-CAN-ACT (intervention protocol developed by the ACTHealthy Lab of the University of Cyprus, implemented for breast cancer patients) protocol [27], in enhancing PTG among cancer patients, compared to standard care, as measured by the Post-traumatic Growth Inventory (PTGI) [28], with efficacy indicated by statistically significantly higher PTGI scores in the intervention group. To comprehensively understand the therapeutic impact and mechanisms behind PTG, several secondary objectives will be approached, including assessing illness perceptions with the Brief Illness Perceptions Questionnaire (BIPQ) [29], where lower scores reflect more adaptive perceptions, Spiritual well-being with the Functional Assessment of Chronic Illness Therapy-Spiritual Well-being (FACIT-Sp) [30,31] where higher scores reflect greater well-being, and clinical symptomatology with the Hospital Anxiety and Depression Scale (HADS) [32] where lower scores reflect reduced anxiety and depression symptom while the presence of any post traumatic stress will be assesed as well. These outcomes are included to examine how ACT influences PTG and its associated domains, offering insight into both clinical efficacy and underlying therapeutic mechanisms. Salivary cortisol will also be collected via self-testing kits to assess physiological stress responses, as a physiological indicator of stress system regulation. Saliva collection offers a non-invasive, feasible, and participant-friendly method to assess cortisol, providing an objective complement to subjective reports. Incorporating this measure allows for the examination of whether the intervention may contribute to restoring adaptive stress responses, enabling a multidimensional understanding of PTG and supporting the investigation of potential mechanisms (e.g., increased flexibility, reduced distress) as well as broader impacts (e.g., improved well-being, physiological regulation) [33,34].

Sample size calculation

According to a priori power calculations using G*Power 3.134 (Heinrich-Heine-Universität Düsseldorf, Düsseldorf, Germany), a sample size of 30-35 participants for each arm (control vs. intervention) of the RCT is sufficient to obtain 80% power with a significance level of 5% and a medium effect size of 0.25. In order to account for a possible 25% drop-out, 40-50 patients diagnosed with each different cancer type should be included in the sample, amounting to a total of around 120-150 recruited participants. Any expected heterogeneity across the three cancer types could indicate the potential need for larger samples that could strengthen transparency.

Patient recruitment eligibility criteria

Participants will be individuals diagnosed with one of three different types of cancer, namely breast cancer, head and neck cancer, or colorectal cancer. Patients (n=120-150) will be recruited from three oncological departments of public hospitals in northern Greece. The selection of these three types was based on both epidemiological prevalence and clinical relevance for psychological intervention associated with substantial psychological burden, survivorship challenges, and body-related concerns that may influence PTG potential [35,36]. Patients are required to have completed chemotherapy and related hospital treatments to ensure they are in a post-treatment survivorship phase, which is when PTG processes are more likely to occur. This timing also minimizes potential interference from acute treatment-related distress and fatigue, which could confound intervention outcomes. Limiting inclusion to individuals with a diagnosis within the past five years helps capture those who are still in the active survivorship window, when adjustment, meaning-making, and growth are most salient. Including patients diagnosed further in the past may reduce the sensitivity of the intervention to detect change related to recent cancer experience. Participants will be recruited using a consecutive sampling technique, whereby all eligible patients presenting to the participating oncology clinics during the recruitment period will be systematically screened and invited to participate.

All consecutive patients with these cancer types who visit the participating clinics will be screened and asked to participate in the IC-Growth study. Patients will be enrolled if they meet the eligibility criteria shown in Table 1. Patient recruitment will start in July 2025 and is planned to end by November 2025.

**Table 1:** Inclusion and exclusion criteria

Inclusion criteria	Exclusion criteria
1. Adults (≥ 18 years)	Being in active psychological or psychiatric treatment
	Severe psychopathology, like suicidal ideation, bipolar disorder, and psychotic disorders
2. Having a diagnosis of (a) breast cancer, (b) colorectal cancer, or (c) head and neck cancer	Having a severe cognitive impairment
	Being unable to give informed consent
3. Having concluded the planned chemotherapy courses and related hospital admissions	Being unable to speak or read Greek
	Active metastasis
4. Having no more than 5 years since the initial diagnosis	Diagnosed with another type of cancer within 5 years before recruitment
	Having less than one year life expectancy

Randomization

Participants will be allocated randomly in a 1:1 fashion to groups (intervention or control) according to specialized software using a computer-generated number sequence, based on a blocked randomization [37] approach developed by Centre for Research and Technology Hellas (CERTH) [38]. The assignment of patients will be conducted during the enrolment phase without prior knowledge of the enrolling clinicians, to avoid biases. Participants will be randomized within each cancer type to ensure that each ACT group is composed of individuals with similar cancer experiences. This decision was made to support group cohesion and psychological safety, as differences in body image, communication difficulties, and treatment side effects may uniquely influence participants' emotional responses and potential for PTG, making cancer-type-specific groups more therapeutically coherent [39-42]. To ensure allocation concealment, the randomization sequence will be prepared in advance by an independent researcher and placed into sequentially numbered, sealed, opaque envelopes. Envelopes will be opened only after a participant has been deemed eligible and has completed baseline assessments, ensuring that enrolling clinicians cannot foresee or influence group assignment. This procedure minimizes selection bias and maintains allocation integrity throughout recruitment.

Ethics

The protocol has received ethical approval from the Ethics and Deontology Committee of Research of the research institute coordinating the study (registry number 25/10-1, dated October 25, 2023), the scientific and ethics committee of a university hospital of the region (registry number 255, dated May 27, 2024), and the scientific and ethics committee of a general public health hospital (registry number 5589, dated April 18, 2024). For ethical reasons, after the completion of the study, the intervention will also be offered to the control group. This will not be part of the study, and no data will be collected. The interventions will be provided based on the promotion of the well-being of the participants, as it would not be ethical not to receive an effective treatment. Informed consent will be obtained from all participants before enrollment in the study. Possible participants will receive a detailed Participant Information Sheet describing the study’s purpose, procedures, potential risks and benefits, data handling, and the voluntary nature of participation. Participants willing to enroll will be asked to sign a written informed consent form. The process will be conducted in accordance with the ethical principles of the Declaration of Helsinki and EU data protection regulations (GDPR). Copies of the signed consent form will be securely stored in locked cabinets at the host institution, and a copy will be provided to the participant. Data processing, as well as the dissemination and exploitation of the study findings, will be conducted in full compliance with EU data protection laws. Any significant protocol modifications (e.g., changes to eligibility criteria, outcomes, or procedures) will be submitted to the ethics committee for approval and updated on the clinical trials registry (NCT07108517).

Monitoring and oversight

Due to the low-risk, psychosocial nature of the intervention, no formal Data Monitoring Committee (DMC) is required. Oversight will be maintained by the principal investigator and study steering team, who will meet regularly to monitor recruitment, data quality, and adherence to protocol. Any adverse events or protocol deviations will be reviewed and reported in accordance with institutional guidelines.

Implementation

Participants, representing the three cancer types, will be randomly assigned to either control or intervention groups (e.g., breast cancer intervention group, breast cancer control group), based on the randomization process (Registration Number NCT07108517). The control groups will receive standard, usual care without any deviation from current practice, while the intervention groups will participate in an ACT-based group program. 

Following approval by the ethics committees of the host organization (CERTH) and collaborating hospitals, the recruitment process will take place at outpatient oncology clinics of three public hospitals in northern Greece, including departments of surgical oncology, medical oncology, and otorhinolaryngology, where patients attend regular follow-up visits or post-treatment assessments. To ensure adherence, healthcare professionals will engage participants in focused discussions, clearly outlining the benefits, risks, and expected outcomes of the trial. Patients who provide informed consent will then be allocated to either the control or intervention group through the randomization process, ensuring transparent and equitable assignment.

**Figure 1:**
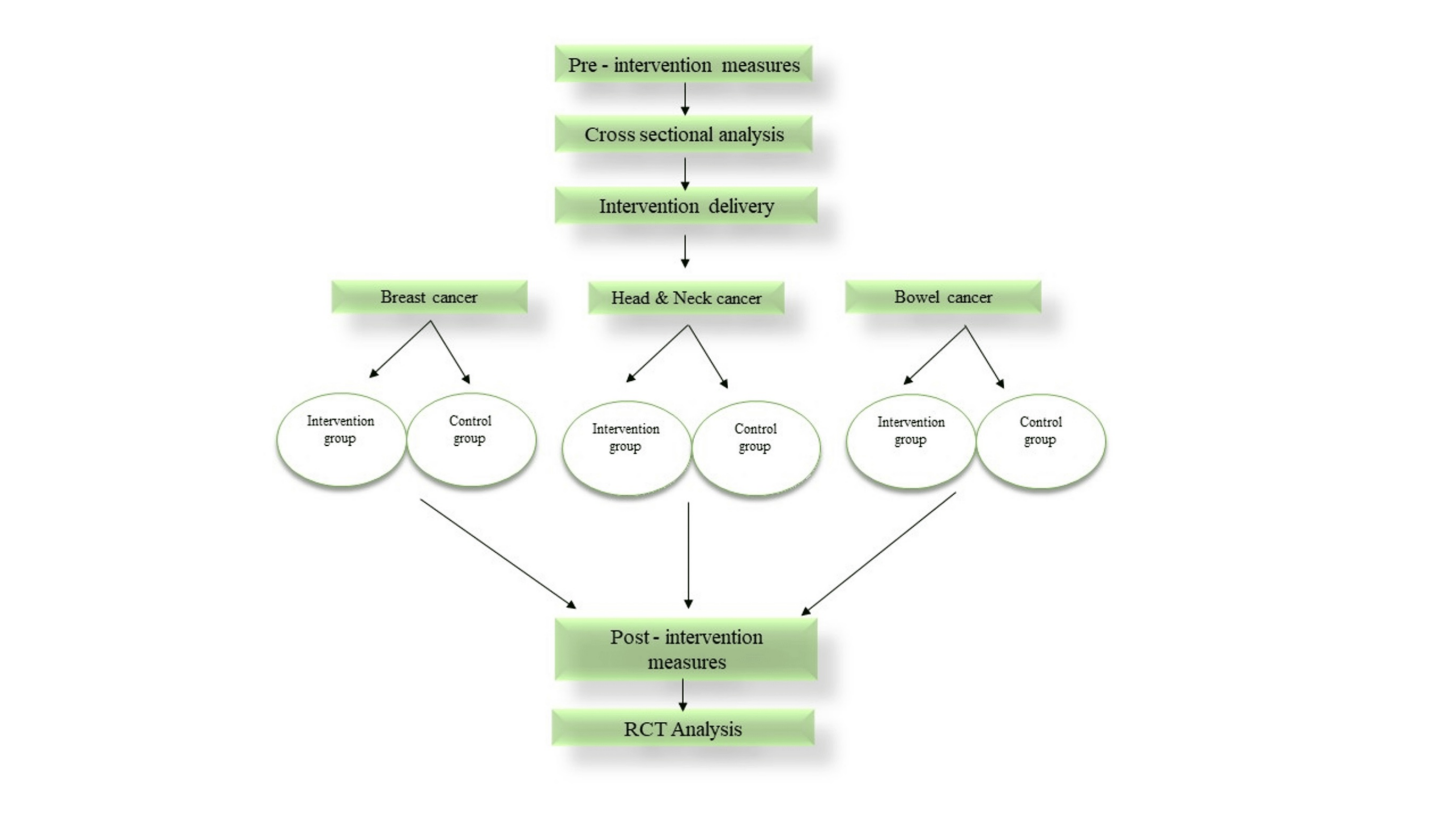
Flowchart of study implementation and analysis RCT: randomized controlled trial

Before the intervention, baseline data will be collected from both groups using the methods described. The intervention will be delivered by two co-therapists in a group format of 8-10 individuals with the same type of cancer. Upon completion of the final intervention session, participants will complete the same evaluation measures used at study entry, while the control group will complete the same measures at the corresponding time (Figure 1).

Intervention group: acceptance and commitment-based group intervention

The research team will deliver and oversee a six-week intervention comprising weekly sessions, each lasting 90 minutes. To effectively address PTG, the intervention employed will be based on an empirically supported psychotherapeutic approach, ACT.

The six-session ACT-based intervention protocol employed in this study was initially designed and developed by Zacharia and Karekla (2021) [27] to address the needs of the I-CAN-ACT project, aiming to improve life outcomes, psychological difficulties, and physical pain in women with breast cancer. Using the I-CAN-ACT protocol, the primary aim is to improve well-being by empowering individuals to live daily life in line with their values and view the self as an arena of experiences, including the experience of cancer [15,27]. Sessions are delivered based on ACT principles, focusing on values, acceptance, cognitive defusion, mindfulness, self-as-context, self-compassion, and committed action [27]. Briefly, the structure of the six sessions is described in Table 2.

**Table 2:** Session structure and content

Session	Theme	Content
Session 1	Introduction and values exploration	Introduction to ACT and learn to identify personal values through exercises that explore the limitations of experiential control and avoidance, as well as the importance of values-driven action.
Session 2	Acceptance and willingness	Focuses on accepting difficult emotions and differentiating between pain and suffering, utilizing exercises such as mindfulness.
Session 3	Cognitive defusion and self-as-context	Teaches distancing from distressing thoughts and introduces the concept of an observing self, separate from thoughts.
Session 4	Values-based goals and committed actions	Participants refine values, set aligned goals, and practice overcoming obstacles to consistent action in line with values.
Session 5	Commitment, self-compassion, and self-as-context	Explores commitment to action and introduces self-compassion practices to manage setbacks, reinforcing resilience.
Session 6	Relapse prevention and life beyond therapy	Concludes with relapse prevention strategies, reviewing key ACT principles for sustainable values-driven living.

Each 90-minute session will follow a consistent structure combining brief didactic instruction, experiential exercises, group discussion, and home practice review. Sessions will introduce each one of the ACT processes (e.g., acceptance, cognitive defusion, values clarification) through short didactic segments, interactive discussions, and experiential exercises. Participants will be invited to share reflections and explore how the concepts relate to their cancer experience. Each session will conclude with a group discussion, a summary of key points, and the assignment of home practice exercises aligned with that week’s theme. This intervention fosters psychological flexibility, encouraging participants to live in accordance with their values despite emotional or physical discomfort [43].

The intervention will be delivered by three licensed psychologists, trained in psychotherapy and experienced in group-based interventions. All therapists have completed or will complete foundational training in ACT and will receive protocol-specific training from the I-CAN-ACT developers before the trial begins. Each group will be co-facilitated by two therapists. To ensure intervention fidelity, selected sessions (1st, 3rd, 6th) will be reviewed using the ACT Fidelity Measure (ACT-FM).

Treatment adherence will be monitored by tracking session attendance, recorded by the group facilitators at each session. Participants attending at least four out of six sessions will be considered treatment-adherent for per-protocol analyses. They will be encouraged to complete home practice exercises between sessions. While these are not mandatory, participants will be invited to briefly reflect on their experiences during the following session. Completion of home practice will be recorded informally to assess engagement with key ACT processes. Drop-outs and reasons for non-completion (when known) will be documented and analyzed descriptively. In cases of missed sessions, participants will be invited to attend a make-up session where feasible, or receive a session summary by phone or email.

Data collection and types of data

The research team will collect data at two separate time points: before the intervention(baseline), and after its completion in both groups. Self-report measures will be collected on paper during in-person sessions and checked for completeness. Data will then be digitized and double-entered into a secure database to ensure accuracy. All physical forms will be stored in locked cabinets at the host institution and securely shredded after digitization, in accordance with General Data Protection Regulation (GDPR) guidelines. Data will include the following: (i) demographic (i.e., age, gender), (ii) clinical Information, including disease and treatment-related questions, and (iii) biomarkers (i.e., cortisol levels) that will be measured by collecting saliva samples by specialized staff.

Assessment Questionnaires

Post-traumatic Growth Inventory (PTGI): PTGI [28,44,45] is a 21-item measure that assesses the personal growth a person experiences after a traumatic event based on five dimensions: relating to others, new possibilities, personal strength, spiritual change, and appreciation of life. Respondents rate each item on a 6-point Likert scale (0 No Change - 5 Change to a great degree). Scores can be interpreted either as continuous variables or grouped into meaningful categories. Higher scores indicate higher PTG (Internal Consistency - Cronbach’s alpha α = .90).

Brief Illness Perceptions Questionnaire (BIPQ): BIPQ [29,46] is a 9-item measure assessing perceptions about patients’ condition, including its consequences, duration, control, and emotional impact, providing insights into how individuals view their illness and its effects on their lives. Items 1-8 are rated on 0-10 numeric scales, and Item 9 is an open-ended causal item. Items 3, 4, and reflect positive perceptions and therefore must be reverse-scored. Higher total scores refer to more negative illness perceptions (Internal Consistency: typically, α = .65-.75 for the overall scale).

Functional Assessment of Chronic Illness Therapy-Spiritual Well-being (FACIT-Sp): FACIT-Sp [30,47] is a 39-item questionnaire that assesses spiritual well-being in individuals with chronic illness. It includes two subscales: Meaning/Peace (assessing the sense of meaning, purpose, and inner peace) and Faith (measuring the role of faith or religion in coping). It captures a broad, non-denominational sense of spirituality and meaning. Each item is rated on a 5-point Likert scale (0 = not at all, 4= very much). Reversed score = 4 - original score. Higher scores indicate high spiritual well-being (Internal Consistency α = .81-.88).

Impact of Event Scale-Revised (IES-R): IES-R [48,49] is commonly used to measure subjective distress caused by a traumatic event in cancer survivors. The subjective impact of the traumatic event can influence the degree of post-traumatic development [28]. The IES-R is a valid 22-question instrument to measure an individual's response to a specific trauma. The questionnaire contains three subscales: avoidance, revival, and overstimulation. Each item is rated 0 (not at all) - 4 (extremely). Higher scores indicate greater post-traumatic distress (Internal Consistency - overall scale: α = .95).

Hospital Anxiety and Depression Scale (HADS): HADS [32,50] can be used to measure anxiety and depression, with higher scores indicating more difficulties. It is a 14-item scale that uses a 4-point Likert scale and has been widely used in research with people with cancer [51]. Each item scored 0-3. For both HADS-A and HADS-D, the score interpretation is the following: 0-7 (no clinically significant symptoms), 8-10 (“possible case”), 11-21 (“probable case” of anxiety/depression) (Internal Consistency: HADS-A: α = .80-.93, HADS-D: α = .70-.90).

ACT-Related Questionnaires

Acceptance and Commitment Therapy-Fidelity Measure (ACT-FM): The ACT-FM [52,53] was developed to measure therapist fidelity to ACT principles. Ensuring treatment fidelity is crucial in clinical trials and practice to confirm that therapy is delivered as intended. The ACT-FM consists of 25 items grouped into four sections based on the Tri-Flex model (Openness, Awareness, Engagement) and Therapist Stance. Items assess both ACT-consistent and inconsistent behaviors. Each item is scored on a 7-point Likert scale: 0 = Not at all evident/not present, 5-6 = High, consistent, skillful demonstration of the ACT process. Higher scores indicate higher fidelity to ACT and higher skill. The measure showed moderate to excellent inter-rater reliability (Internal Consistency Cronbach’s alpha -total scale: α = .91) and was considered usable across different therapy contexts.

Acceptance and Action Questionnaire-II (AAQ-II): The AAQ-II [54,55] is a 7-item self-report measure that evaluates psychological inflexibility and experiential avoidance, core constructs in ACT. The AAQ-II captures an individual's unwillingness to experience distressing thoughts and emotions and the extent to which these experiences interfere with valued action. It has been used widely in both clinical and non-clinical populations. Higher scores reflect greater experiential avoidance and lower psychological flexibility. Each item is rated on a 7-point Likert scale (0 = never true, 7= always true).

Valued Living Questionnaire (VLQ): The VLQ [56,57] is designed to assess the degree to which individuals live by their personally chosen values, a central focus in ACT. It is a two-part self-report measure: the first part evaluates the importance of 10 life domains (e.g., family, work, health), and the second assesses how consistently individuals have lived in alignment with their values over the past week. A composite score is calculated by multiplying importance and consistency ratings for each domain. Each scale demonstrates good psychometric properties, including satisfactory internal consistency, test-retest reliability, and construct validity. Each of the 10 domains is rated on a 10-point scale: 1 = Not at all important/ consistent, 10 = Extremely important/ consistent. Higher composite scores mean greater valued living (values clarity + actions in alignment). All measures are translated, culturally adapted, and psychometrically validated for use in Greek-speaking populations, confirming their suitability for application in Greek clinical settings. Consequently, these instruments are deemed appropriate for inclusion in the proposed clinical trial.

Data security

All sensitive information will be stored in accordance with the General Data Protection Regulation of the European Union (GDPR). The responsible Research Center is also ISO-certified for GDPR compliance. Bioethics approval has been obtained. Anonymity will be maintained at all stages of the data-handling and sharing process. Any information that could identify participants will be anonymized, and each participant will be assigned a unique identification code. A password-protected document will link participant details with their respective code. Personalized data will be retained for five years after the study's completion. Handwritten data (e.g., questionnaires) will be digitized as soon as possible and then securely destroyed using a shredder.

Digital data will be stored on password-protected external hard drives. Saliva samples for cortisol measurement will be collected by trained staff at two time points: pre-intervention (baseline) and post-intervention (follow-up). Participants will be instructed to avoid eating, drinking, or brushing their teeth at least 30 minutes before sampling. Samples will be immediately refrigerated and securely stored at CERTH facilities, labeled only with pseudonymized codes and no personal identifiers. They will then be analyzed on-site, and results will be promptly digitized and transferred between CERTH’s secure email accounts. After analysis, all biological materials will be sealed in biohazard bags and safely destroyed. The research team will not have access to participants’ full medical records; instead, treating physicians will share only the necessary clinical information with the researchers in compliance with GDPR procedures.

Primary and secondary data analysis plan

Normality will be assessed using the Shapiro-Wilk test and inspection of skewness and kurtosis. Homogeneity of variance will be tested with Levene’s test. Between-group comparisons will be conducted with Mann-Whitney U tests, within-group changes with Wilcoxon signed-rank tests, and group-by-time effects with non-parametric repeated-measures tests (e.g., Friedman test or aligned-rank transform procedures). All statistical analyses will be conducted using appropriate software (e.g., R, v. 4.3.2, R Foundation for Statistical Computing, Vienna, Austria, https://www.R-project.org/; IBM SPSS Statistics, v. 27, IBM Corp., Armonk, NY, USA). Data screening will include identifying and addressing outliers, as well as testing the assumptions of the statistical tests employed. Questionnaire data will be analyzed using Cronbach’s alpha to ensure reliability and validity within this specific population. Descriptive statistics will be provided for demographic (gender, age group, origin, etc.) and clinical characteristics (diagnosis, disease stage, etc.) recorded at baseline. The primary outcome, PTG, will be analyzed using mixed-design ANOVA or MANOVA, comparing pre- to post-intervention changes between the intervention and control groups. The primary analysis will be conducted on the total sample, combining cancer types, as the trial is powered at the overall group level. This approach is justified by the transdiagnostic nature of ACT, which targets psychological processes common across cancer types (e.g., psychological flexibility, values-based action). Exploratory stratified analyses will also be conducted to examine whether cancer type moderates the intervention’s effects. Secondary outcomes-including psychological distress (HADS), illness perceptions (BIPQ), spiritual well-being (FACIT-Sp), and ACT process measures (AAQ-II, VLQ)-will be analyzed using similar pre-post mixed ANOVA or MANOVA models, with appropriate corrections for multiple comparisons (e.g., Bonferroni).

Biomarkers will be analyzed using repeated measures MAN(C)OVA, assessing changes from baseline to post-intervention between groups. Non-normal data will be log-transformed. Skewed continuous variables (particularly biomarker data such as cortisol) will be log-transformed using the natural logarithm to reduce skewness and stabilize variance. Analyses will control for potential covariates such as age, sex, and cancer type. In addition, correlational and exploratory mediation analyses may be used to examine associations between biomarker changes and psychological outcomes, thereby exploring potential biopsychosocial mechanisms of PTG. Bonferroni corrections will be applied for multiple comparisons. As for the cross-sectional data, correlations and network analysis will be conducted to examine interconnected relationships among variables. Statistical significance will be set at p < .05 (two-tailed).

Data sharing plan

De-identified participant data and statistical code will be made available upon reasonable request to the corresponding author following publication of the trial results, in accordance with institutional and ethical guidelines.

## Results

Safety reporting

The research does not involve physical, legal, or social risks. However, about the psychological dimension, some of the questionnaires include sensitive items (e.g., quality of life), and participants may be asked to discuss difficult topics (e.g., their diagnosis), which could cause temporally psychological discomfort. To minimize the likelihood of psychological distress, individuals undergoing active treatments for their condition will not be approached. Additionally, those receiving ongoing psychological support will be excluded from participation. The researcher's contact information will be provided to all participants in the information sheet to offer further support should any difficulties arise from participation in this study. During the intervention, if any of the participants show high levels of distress, they may withdraw from the study, and a psychologist will provide Psychological First Aid. Such participants will be referred to individual therapy and withdrawn from the group for the remainder of the intervention. Currently, three intervention and three control groups have been completed. The recruitment process continues to complete the necessary number of participants for both groups.

## Discussion

Despite the growing recognition of post-traumatic growth (PTG) as a significant psychological outcome among cancer patients [5], there remains a need for clinical trials to assess the efficacy of interventions aimed at fostering PTG. To date, much of the literature has relied on observational and cross-sectional studies, which, while informative, do not allow a clear understanding of how PTG develops over time or which factors most effectively promote it [58]. Preliminary research suggests that therapeutic interventions hold promise in facilitating PTG [6,11,59,60]; however, these findings require validation through controlled trials. Such studies provide a unique opportunity to evaluate the efficacy of these interventions and to elucidate the mechanisms through which PTG can be cultivated and sustained in cancer patients. The clinical trial reported herein seeks to evaluate the efficacy of an ACT-based group intervention in fostering PTG among cancer patients. Addressing the current scarcity of intervention protocol studies in this area, the trial employs an experimental longitudinal design with validated outcome measures to rigorously assess the intervention’s effectiveness.

If effective, this study could provide robust evidence for ACT as a targeted intervention to enhance posttraumatic growth in cancer survivors, while clarifying the psychological and physiological mechanisms that contribute to positive adaptation after cancer. The I-CAN-ACT protocol is also well positioned for scalability within public oncology settings, as its structured, group-based format can be delivered by trained clinicians with modest resource requirements. Together, these features highlight the potential for integrating ACT-informed, mechanism-based psychosocial care into routine survivorship pathways to improve patient well-being on a broader scale. By filling this research gap, the trial aims to advance understanding of the processes underlying PTG and contribute to the development of targeted, evidence-based therapies that can be integrated into clinical practice to enhance psychological resilience and well-being in cancer care. clinical practice to enhance psychological resilience and well-being in cancer care.

Study strengths and limitations

This study has several notable strengths. It employs a randomized controlled design for evaluating psychosocial interventions and allows for robust causal inferences. The trial integrates multidimensional outcomes-including psychological measures, ACT process variables, and physiological biomarkers-providing a comprehensive assessment of both efficacy and underlying mechanisms. The use of validated, culturally adapted Greek questionnaires enhances the precision and relevance of measurement. Additionally, the intervention is based on a well-established ACT protocol delivered by trained clinicians, with fidelity monitoring to ensure consistent implementation. Recruiting participants from multiple public oncology centers further strengthens the external validity of the findings across diverse cancer populations.

While this trial aims to address critical gaps in understanding how therapeutic interventions can foster post-traumatic growth (PTG) among cancer patients, limitations should be noted. The study will be conducted across oncology centers within a specific region of Greece, which may affect the generalizability of the findings to other populations or healthcare systems. In addition, the study primarily relies on self-reported psychometric instruments, which, although validated and widely used, are susceptible to subjective bias. Finally, the six-week intervention period allows for the assessment of short-term effects may capture the sustainability of PTG for a specific period of time. So, after the completion of this study, extended follow-up periods and broader samples could verify and expand the findings.

## Conclusions

This study protocol outlines a randomized controlled trial evaluating the efficacy of an ACT-based group intervention (I-CAN-ACT) in enhancing posttraumatic growth (PTG) among breast, colorectal, and head and neck cancer survivors. By targeting psychological flexibility through a structured, transdiagnostic intervention, this trial aims to address a critical gap in psycho-oncology: how to support positive psychological adaptation following cancer. In addition to PTG, the study incorporates a multidimensional set of secondary outcomes, including distress, spiritual well-being, illness perception, and biological markers of stress, to explore potential mechanisms of therapeutic change. Through rigorous methodology, validated measures, and attention to implementation factors, this research seeks to generate actionable insights into the development of evidence-based psychosocial interventions. If effective, the intervention could offer a scalable model for enhancing psychological resilience and quality of life in cancer survivorship care.
